# Topics of the Month

**Published:** 1894-10

**Authors:** 


### TOPICS OF THE MONTH.

SINCE the University of the State of New York, by virtue of the medical practice act, has assumed control of the examinations for license to practise medicine in this state, new interest attaches to its organization as well as the details of its work. Physicians all over the country are inquiring into its methods, and foreign governments are soliciting its reports. We have heretofore, on a few occasions, briefly alluded to the advantages it is affording physicians and students by the methods it has adopted for preliminary as well as final examination.

The following, from the *New York Polyclinic*, Sept. 6, 1894, is cheerfully reproduced in view of the facts above referred to:

> What is the University of the State of New York? While it holds the "futures," so to speak, of all the embryo doctors, as well as the graduates of every medical college under the recent law, how few could answer the question. Yet the question is well worth answering, for under that title we possess an institution of remarkable administrative capabilities, of utility which, if not absolutely unique, is only paralleled in France, and approached within a certain distance by the London University.
>
> The history of the University of the State of New York is bound up with that of the commonwealth, with which it is nearly coeval. Organized in 1784, it had for its first chancellor George Clinton, while DeWitt

Clinton was its third secretary ; and Alexander Hamilton and John Jay were among its first and most active regents. The "University" is a supervisory and administrative, not a teaching, institution. It is a state department, and at the same time a federation of over 500 institutions of higher and secondary education. Like other states, New York has a department of public instruction in charge of elementary schools, but no other state has a department devoted to the interests of education higher than that which may be classed as elementary.

Considered as a state department, the "University"—we use quotation marks to distinguish it from the meaning usually attached to the term—unites various educational functions elsewhere scattered or entirely unprovided for, and exercises unusual powers. Though most of its work is executive, yet in granting charters to all educational institutions it performs functions usually discharged only by legislatures. On the other hand, in revoking charters and dissolving educational corporations, it exercises the judicial functions of a court. Its examination department embraces not only the functions of the local examinations of Oxford and Cambridge, and of the University of London, but also the state licensing of physicians, and other work peculiar to itself. Its extension department corresponds to the similar departments of Oxford and Cambridge, and of the London Society for the Extension of University Teaching. Its state library and state museum departments not only have custody of collections, among the most important in the United States, but also conduct work allied to that of the English science and art department of the British Museum, and of the London natural history museum.

Regarded as a federation, the University of the State of New York reminds one of the Universities of Oxford and Cambridge, each of which represents a union of colleges ; but in respect of comprehensiveness it differs from these, because it includes all the colleges, academies and institutions for higher education within the bounds of the commonwealth. The law gives to incorporated institutions no option as to their membership in the University. It says : "The institutions of the University shall include all institutions of higher education (the term 'higher' here is construed to embrace what is generally termed secondary) which are now, or may be hereafter, incorporated in this state, and such other libraries, museums, or other institutions for higher education as may, in conformity with the ordinances of the regents, after official inspection, be admitted to or incorporated by the University." This means that no educational establishments, except those of a relatively elementary order, which fall under the authority of the department of public instruction, can have a legal existence in New York without being subject to the regulations and entitled to the privileges provided by the University.

The University of the State of New York conserves the advantages of individual initiative, while extending the benefits of system, of harmony, of co-working in the various members of the educational body, and of organic connection with state life. The organization most closely resembling it is that of the University of France, as devised by the first Napoleon in 1808, twenty-four years after that of the State of New York had been put in operation.

---

IN A RECENT number of the JOURNAL we had occasion to refer to the fact that an attempt was made last Winter in the legislature to reduce the State examining fees for license to $5.00, which, if adopted, would practically wipe out the State examining boards. Happily, however, this was defeated. We suggested, at that time, that it would be more rational to reduce the college fees for diplomas to $5.00, which would be adequate to cover the expenses connected with issuing a certificate of study.

In the *Journal of the American Medical Association* we find another plan proposed to relieve students from alleged hardships relating to advanced medical education, as follows :

In an address on higher medical education, before one of our State medical societies, the orator dwells on the alleged hardship to the student when he fails in his finals, and says : "If the law compels a young man to study four years, it ought to give him some guarantee that he will be fairly dealt with. Were our medical schools compelled to refund one-half the expenses of the student who fails to get his diploma (*i. e.*, one-half the cost of three years' maintenance and of three years' loss of time), if the schools were obliged to refund all moneys expended on lectures, the graduating class would not be reduced in numbers, but the 10 per cent. that fail at their final examinations would have some redress." This is just the kind of a proposition that would be apt to strike favorably a certain class of legislators, and it would not be in the least surprising to see it, in the near future, embodied in "An act entitled an act," etc. Meanwhile, the *Journal* commends, to the author of the address under consideration, the following cold facts which have already appeared in these pages : In round numbers, there are upwards of 118,000 doctors in this country, which, with a population, also in round numbers, of 65,000,000, gives an average of one doctor to every 550 men, women and children ; this population increases at the rate of about 2.5 per cent. per annum, but every year we add over 5 per cent. of new doctors. It does seem that we might worry along without making it any less "hard and unsatisfactory" to become a doctor, for the present, at least.

THE *North Carolina Medical Journal*, in a recent number, comments on the ignorance of midwives as follows :

The ignorant, depraved and filthy women who style themselves as midwives in North Carolina, are a disgrace and a shame to nineteenth century civilization. The fate of the parturient woman in heathen India, who is shut up by herself until she has passed through the throes of labor, is no worse, and in many cases better, than the unfortunate one who falls into the hands of some of these creatures. That there should be midwives, who are capable of conducting women through "the perils of child-birth," we suppose is a necessity now, and will be for long years to come, but that this duty should be entrusted to any old ignorant negro, who takes a notion to be a "granny," is supremely wrong.

This class, who deal with the lives of two beings at once, should be controlled by the strictest laws. They should be required to have their names and residences registered with the county clerk, and he should be allowed to register them only when they present a certificate from some qualified person—the county superintendent of health, for instance—showing they possess a reasonable degree of knowledge in the management of cases of labor, including asepsis, diagnosis of presentations, placenta previa, causes for unusual delay in delivery, and proper care of mother and child after labor. They should be required to summon the aid of a registered physician as soon as any abnormal condition is detected—in other words, they should be permitted to conduct to their close only normal cases of labor. They should not be permitted to administer drugs to their patients, and especially ergot, unless all the contents of the uterus have come away. We notice in one of the Northern States (New York, we think), in an act regulating the practice of midwifery, midwives are prohibited from giving drugs *except ergot*. This is probably the most dangerous drug that could have been left in their hands.

---

PRIVATE hospitals are so common nowadays as not to attract any special notice either from the profession or laity. Almost every small city has at least one, and in large cities their number is not easily ascertained without the aid of the fundamental principles of arithmetic. It is a fashion that began in the East, but is having its full sway in the West at present.

From this foundation has grown up another system, in which corporations have established so-called sanitaria, issuing stock to physicians whose names appear on the announcements either as attending or consulting members of the staff. It is not our purpose to criticise the establishment of private hospitals for the treatment of surgical or gynecological cases by scientific physicians. The advantages of this plan are too well understood by the profession and by the patients themselves to need any extended argument to prove them or to admit of any adverse criticism.

But we do think that these great sanitarium companies, established in small towns and cities throughout the country, are overdoing the work most decidedly. A reaction is sure to follow the sanitarium craze that has now taken possession of the semi-invalid and the incompetent doctor.

———

THAT the injection of glycerine, to induce premature labor, is not entirely free from danger, has been frequently demonstrated. The *Richmond Journal of Practice*, August, 1894, contains the following note on the subject that will be found of interest:

Since Pelzer announced that glycerine, when injected between the uterus and fetal sac, would induce premature labor, it has generally been regarded a safe measure, provided everything is absolutely sterile, and the precaution is taken to avoid forcing air through an empty catheter, by filling the catheter, of course, before using it.

That glycerine is not always free from danger has been shown by Pfannenstiel, in an article published in the *Centralblatt für Gynecologie*, No. 4, 1894. Dr. Oscar Embden, of Brooklyn, reports also a case with similar effects in the *Medical Record*, July 28th, and refers to Pfannenstiel's two cases. In two of these cases there were unmistakable symptoms of glycerine poisoning, chief among which was a nephritis associated with hemoglobinuria—*i. e.*, the presence of the red coloring matter of the blood in the urine. This coincides with experiments on dogs and rabbits, the decomposition of the blood producing first a glomerulo-nephritis, which is followed after the injection of more glycerine by interstitial nephritis as well as interstitial hepatitis.

In one of the cases reported there was slight nephritis before the glycerine was injected into the uterus, and this was, of course, intensified. It would seem, therefore, according to these reports, that Pelzer's method is not absolutely devoid of danger, and hence should not be indiscriminately used.

———

THE antitoxin treatment of diphtheria is attracting considerable attention, especially in Great Britain, where reports are continually accumulating of its benefits. In the *British Medical Journal*, September 8, 1894, is an editorial, from which we abstract the following:

The earliest published cases of diphtheria treated by this method, were a series of thirty reported by Behring and Kossel, in the *Deut. Med. Woch.*, April 27, 1893. Of these, twenty-four, or 80 per cent., recovered. In April, 1894, Ehrlich, Kossel and Wassermann, in the same journal, reported the results in 220 unselected cases of diphtheria, treated by the hypodermic injection of the serum of goats rendered immune by giving them increasing doses of dead diphtheria cultures. Among the 153 cases, which it was not necessary to submit to tracheotomy, the mortality was only 23.6 per cent. In six treated on the first day there was no death, and in sixty-six treated on the second day there were recoveries amounting to 97 per cent.; whereas, in twenty-three treated on the fourth day the percentage of recoveries fell to 56.5 per cent. It is important to add that in a few instances the great improvement noticed in the first two days was not maintained, and the patients died in ten days or a fortnight of nephritis or cardiac failure. There is some evidence that a person may be rendered immune to the infection of diphtheria by preventive inoculations. Guinea-pigs have been rendered immune by inoculation with the blood serum of patients just recovering from the disease. The dose, to be injected as a prophylactic in persons liable to be exposed to diphtheria, is set down by Behring at sixty antitoxin normals, or one-tenth of number one. After infection, that is, during the incubation stage, he believes that a 150 antitoxin normals ought to avert the development of the disease. Behring states that the serum, prepared and tested under his own supervision, is issued in two forms, number one and number two. Number two is two and one-half times stronger than number one. Number one is sufficient for the treatment of diphtheria in a child under ten years of age. If it be seen on the second or third day, number two serum acts more surely and rapidly. The estimation of the exact strength of the serum is a difficult matter, and it must be recognized that the strength is liable to vary with the commercial source from which it is obtained.

